# Indonesia free from pasung: a policy analysis

**DOI:** 10.1186/s13033-023-00579-6

**Published:** 2023-05-03

**Authors:** Muhamad Taufik Hidayat, Candice Oster, Eimear Muir-Cochrane, Sharon Lawn

**Affiliations:** 1 College of Medicine and Public Health, Flinders University, PO Box 2100, 5001 South Australia, Adelaide, South Australia Australia; 2 West Java Psychiatric Hospital, Bandung, Indonesia; 3 College of Nursing and Health Sciences, Flinders University, PO Box 2100, 5001 Adelaide, South Australia Australia; 4 Lived Experience Australia Ltd, Adelaide, Australia

**Keywords:** Pasung, Policy analysis, Mental Health, Seclusion, Restraint, Community Care

## Abstract

**Background:**

Many people with mental illnesses remain isolated, chained, and inside cages, called Pasung in Indonesia. Despite numerous policies introduced to eradicate Pasung, Indonesia has made slow progress in decreasing this practice. This policy analysis examined existing policies, plans and initiatives in Indonesia targeted at eradicating Pasung. Policy gaps and contextual constraints are identified in order to propose stronger policy solutions.

**Methods:**

Eighteen policy documents were examined, including government news releases and organisational archives. A content analysis was undertaken of national-level policies that address Pasung within the context of the health system, social system and human rights since the establishment of Indonesia. This was followed by a case study analysis of policy and program responses particularly in West Java Province.

**Findings:**

While policy to address Pasung exists at a national level, implementation at national and local levels is complicated. Pasung policy has generated a sense of awareness but the different directions and ambiguous messaging across all stakeholders, including policy actors, has created a lack of clarity about institutions’ roles and responsibilities in the implementation process, as well as accountability for outcomes. This situation is exacerbated by an incomplete decentralisation of healthcare policymaking and service delivery, particularly at the primary level. It is possible that policymakers have overlooked international obligations and lessons learned from successful policymaking in comparable regional countries, resulting in disparities in target-setting, implementation mechanisms, and evaluation.

**Conclusion:**

While the public has become more informed of the need to eradicate Pasung, ongoing communication with the various clusters of policy actors on the aforementioned issues will be critical. Addressing the various segments of the policy actors and their challenges in response to policy will be critical as part of building the evidence base to establish a feasible and effective policy to combat Pasung in Indonesia.

**Supplementary Information:**

The online version contains supplementary material available at 10.1186/s13033-023-00579-6.

## Introduction

Human rights violations against people with mental illnesses are widespread in both community settings and mental health facilities in many countries [1]. One example of violations is the use of seclusion and restraint (SR). Human Right Watch reported that at least 60 countries have SR practices [2] in their mental health institutions and communities. Inpatient mental health facilities use SR as a method of management for the safety of psychiatric patients and others [3], yet they are not therapeutic measures and should only be used as a last resort [4]. Although SR is clinically effective in terms of maintaining safety and administering essential medication, it is typically regarded as a high-risk procedure [5, 6]. They are also typically counterproductive, with harmful repercussions for both patients and mental health professionals because they damage trust and engagement with services and promote coercive care over therapeutic care [5–7]. Seclusion and restraint are also widespread in community settings in Indonesia where it is known as Pasung, and it is frequently used, prompting significant human rights concerns [8–10].

Pasung is a ‘long-standing custom’ in West Java and Indonesia, more broadly. Pasung has traditionally involved a person who is agitated or considered at high risk of doing harm, either to themself or others, being shackled using a wooden log [8, 10, 11]. Frequently, this shackle is anchored to the concrete floor or wall [8, 12]. Currently, Pasung has grown to include purpose-built cages and similar structures within the home or community designed for containment.

In Indonesia, the policy to ban Pasung was issued in 1977 with the release of a Home Affairs Ministry regulation [13]. Despite this regulation being in existence for more than four decades, Pasung still exists in the Indonesian community and has been somewhat overlooked. In 2010, the Indonesian government through the Ministry of Health launched the Indonesia Free Pasung Program, which aimed to eliminate the usage of community SR for people with serious mental illness [14]. It was followed by the ratification of the Mental Health Act.18, which had been in effect since 2014 and reaffirmed that those who deprive others of their rights would be imprisoned or fined [15].

There have been no epidemiological surveys that have accurately expressed the percentage of people in the population subjected to Pasung until recently [10]. In Indonesia, the majority of people with mental health issues are hidden by their families and not exposed to public view. This is because mental illness is highly stigmatised and viewed as a shameful affliction in Indonesian culture and society [8, 16].

The Indonesian government claims that the Free Pasung Program has successfully reduced Pasung based on rates estimated by Human Rights Watch from 18,880 cases in 2010 to 12,220 cases in 2018 [17]. The Indonesian government’s repeated revisions of the Free Pasung Program (2010–2017 and 2019) have sparked doubt among the community about the true number of people still in Pasung and the success of the Free Pasung Program as claimed by the government. The number of individuals in Pasung could be significantly higher than estimated. Furthermore, according to data given by the Indonesian Centre for Health Research, the number of people with serious mental illness who were subjected to Pasung decreased somewhat from 14.3 to 14%, but this does not match the fall in rates stated by the Ministry of Health. It also doesn’t appear to reflect Indonesia’s population growth from 2010 to 2018, when the country’s population increased by about 30 million (from 237 to 265 billion) [18]. According to data from the Ministry of Health website, 10% of those in Pasung were released and treated in hospitals over a six-year period between 2009 and 2014. However, there is no evidence on how many of those people with serious mental illness were successfully rehabilitated or returned to their communities and returned to Pasung (see Additional file 1).

Since the introduction of the Pasung policy, there has been limited research into how the policy has been positioned to bring about changes and what the policy actors perceive to be the challenges [9, 19–21]. Among this research, some are focused on the content [20, 21] and other on actors and decision-making [16]. The political, economic, and social contexts in promoting or inhibiting Pasung policy at the national and subnational levels are largely unexplored.

Using Health Policy Analysis (HPA) [22], otherwise known as the Policy Triangle Framework (PTF) [22, 23], this policy analysis intends to contribute to addressing this knowledge gap. This framework has been widely used in many countries, particularly low- and middle-income countries, to address a variety of health policy issues, including health sector transformations and population health [24–26]. It focuses on four key aspects: the policy’s content; the people involved in policy change; the procedures involved in producing and implementing change; and the context in which the policy is created. The framework is premised on the idea that policy derives from and is shaped by political and social processes [22, 26, 27].

The HPA is a simplified portrayal of a complicated set of interrelationships in which actors (as individuals or members of groups or organizations) are influenced by the context in which they live and work; context is influenced by many factors such as political instability or ideology, history, and culture; and the policy-making process – how issues get onto policy agendas and how they fare once there – is influenced by actors and their position in power structures [25].The context variables that have shaped Pasung policy, the actors involved, the content of the policy and institutional provisions and the approaches and policy processes are examined in this study. The findings of this study will be used to inform the challenges and accomplishments to the change agents, such as relevant government agencies, and will contribute to the body of knowledge on Pasung, hence strengthening the links between research and policy.

## Methods

### Sampling frame of included literature

All policies that aimed to address Pasung, whether in English or Indonesian, with full text available and have been issued by the government of Indonesia from 1945 onwards, including the Indonesian Constitution, laws related to the right to health in Indonesia, human rights, and social welfare for people with mental illness subjected to Pasung were included. For the availability of targeted Pasung policy documents and publications, we mostly relied on online resources, searching the websites of the Ministry of Health, Ministry of Social Affairs, Ministry of Home Affairs, Ministry of Law and Human Rights, Human Rights commission report, and the West Java Provincial Government.

### Search Strategy

Web-based searches of national and provincial websites, including the Ministries of Health, Ministries Social Affairs, Ministry of Law and Human Rights, and Ministry of Home Affairs, the Human Rights Commission report, and the West Java Government website, were conducted to identify all public policy documents relevant to Pasung. Searches were conducted in in September 2021. All potentially relevant information was downloaded for analysis. Search terms included “Pasung”, “mental illness”, “mental health”, “policy development”, “policy implementation”, “policy evaluation”, “disability”, “Pasung policy”, and “health policy”. We included 17 national policy documents and one provincial level policy document in our analysis.

### Data extraction and analysis

We began by identifying, describing, and categorising current and previous policies aimed at overseeing Pasung practice. We compiled and reviewed the content of all national policies dating back to 1945, looking for particular policy content pertinent to Pasung and then went on to analyse and explain the reasons for their impact (or lack thereof) on Pasung. We tracked the evolution of policy content over time, as well as the extent to which the above-mentioned shifting policy framework influenced implementation.

The analysis then moved to the provincial level where the study was located which is West Java Province’s health policy and strategy papers. Understanding how decentralisation reforms impacted policy creation and execution, a review of provincial Pasung prevention plans and implementation policies was considered relevant. West Java was chosen because it is one of the provinces with the highest prevalence of mental health problems according to National Health Research in 2013 [28]. Despite a minor drop in 2018 (using a different instrument compared to the previous one in 2013), West Java is also regarded as having among the highest number of people in Pasung in Indonesia, given the fact that it is the most populated province in the country [18, 29].

The context, content, mechanisms, and actors that shaped these policies were examined using the HPA. The term “context” refers to national, regional, or even worldwide political, economic, social, and cultural elements that may influence health policy. The reviewed policy’s content refers to what areas of healthcare it covers and what is not covered. The mechanisms through which these policies were formed, implemented, or reviewed is referred to as the policy process. Individuals, communities, groups, institutions, and the government are examples of actors who have an impact on health policy. Lastly, the data was read for familiarisation, then iteratively read again to discover any new trends. Context, actors, content, and processes were among the key categories of codes examined and classified based on Walt and Gilson’s established codes and themes [22, 25].

## Results

### Policy documents identified

Eighteen policy-related documents were identified. In this section, each document is described in relation to its relevance to Pasung. We divided the context of Pasung policy into three periods of time considering that Indonesia has had three Eras since its independence in 1945. The first Era is called the ‘Old Era’, starting from 1945 to 1965; the Second Era, called the ‘New Order Era’, began in 1966 after the rebellion of the Communist parties in which thousands of civilians and army personnel died, up until 1998; and, the Third Era is called the ‘Reformation Era’, starting from 1999 to the present [30–33]. Table 1 shows the chronology of the policies and plans evaluated for relevance to Pasung.

**Table 1:** The Timeline of Major Policies of Relevance to Pasung

No	The Policy	Year	Era
1	The Constitution of the Republic of Indonesia	1945	First Era
2	The Indonesian Penal Codes (enacted by Law No 1 of 1946)	1946	
3	Mental Health Act	1966	Second Era
4	Ministry of Home Affairs decree of 1977	1977	
5	Law on Health No 23 of 1992	1992	
6	Human Rights Act Number No. 39	1999	Third Era Part 1 - Before the Enactment of Mental Health Act No. 18 of 2014 Part 2 - Global Policy shift from Millennium Development Goals (MDGs) (2000–2015) to Sustainable Development Goals (SDGs) (2015–2030)
7	The amended 1945 Constitution	2000	
8	Law on Indonesia Social Security Scheme No. 40	2004	
9	Law on Health No.36	2009	
10	Towards Indonesia Free of ‘Pasung’ Ministry of Health	2010	
11	Law on Social Security Agency No. 24	2011	
12	Mental Health Act No. 18	2014	
13	Law concerning the Rights of Persons with Disabilities No.8	2016	
14	Ministry of Health Ministerial Decree on Health Indonesia through Family Approach (PIS-PK) No. 39	2016	
15	Stop Pasung movement (Gerakan Stop Pemasungan/GSP)	2017	
16	Ministry of Health Ministerial Decree on Stop Pasung No 54	2017	
17	Ministry of Health Ministerial Decree on Minimum Services Standard on Health No 4	2019	
18	West Java Regional Regulation of mental health	2018	Local policy

### The first era after the Proclamation/Old era (1945–1965)

#### Context

After the proclamation of its independence in 1945, the newly independent Indonesia inherited a health system that had been destroyed by years of combat, including Japanese occupation and revolutionary battle against the Dutch [34, 35]. There was a severe scarcity of clinicians in the country, who were largely concentrated in the country’s major cities, where only a small percentage of the people lived. The Indonesian Ministry of Health also had to manage the recurrence of epidemic diseases as well as endemic diseases [34].

Indonesia adopted a mental health system similar to that of the United States, focused on a clinical biomedical paradigm. American psychiatry dominated mental health treatment from then on, setting the groundwork for modern, open-style institutions and outpatient care. Most Indonesian psychiatrists undertook training at Western universities after independence and applied their expertise of the topic in their own country [36]. Since the mid-1960s, the biomedical paradigm has been the foundation of psychiatric thinking. The medicalization of mental health issues tends to background the patient’s subjective experience of the condition in favour of delivering accessible mental health care within a complicated health system. The Directorate of Mental Health in Indonesia is in charge of this. However, due to the government’s inadequate health infrastructure, people in rural and remote locations have limited access to treatment [36, 37].

#### Content

There were two policies issued during the Old Era which were the 1945 Constitution of the Republic of Indonesia and The Penal Code of 1946. The details are below:

1. **The Constitution of the Republic of Indonesia**
  The 1945 Constitution of the Republic of Indonesia [38], also known as Undang Undang Dasar 1945, is the foundation of all law in Indonesia. It was written before Indonesian independence in August 1945 and was named shortly after that independence was proclaimed, following Dutch colonial rule and then Japanese occupation during World War Two. The constitution was a brief document consisted of 37 articles, six of which dealt specifically with human rights (Articles 26–31) as shown in Table 1. However, only one Article (Article 27 verse two, in bold text) was directly related to Pasung, that:
  Every citizen is entitled to work and a living that is commensurate with their status as human beings.
  It is worth noting that the Constitution’s obligations to human rights predate the 1948 United Nations Universal Declaration of Human Rights. The above rights were to be established by law, and they might also be restricted by law. For example, every citizen has a right to a living that is consistent with their status as human beings, but if they are declared mentally ill, they may be forced to live in a mental hospital as other laws may allow family or community members to transfer someone to the hospital without their consent.
2. **The Penal Code 1946**
  In the Penal Code 1946 [39], three Articles dealt with mental illness and, to a lesser extent, seclusion and restraint. Despite the fact that the penal code prohibits a person from being deprived of liberty, someone who allows a mentally ill person to be abandoned alone might be imprisoned and punished.
  - Indonesian Penal Code Article 333 verses 1.
    Any person who deprives a person of liberty purposely and unlawfully, or proceeds to deprive a person of liberty, faces a maximum sentence of eight years in jail.
  - Article 10.
    For the sake of peace and public order, or to heal the mentally ill person himself, close relatives of a mentally ill person may petition the chairman of the district court to have the person treated in a mental health facility
  - Article 491.
    Anyone who is obligated to care for a mentally ill person who is hazardous to himself, or others faces a maximum fine of 750 rupiahs if they let that person roam around unaccompanied
    This means that a family’s options for caring for someone with mental illness are limited. On the one hand, they are prohibited from depriving a person of their liberty, but on the other hand, they cannot allow the person to roam since they will encounter severe consequences. When the family cannot send this individual for treatment because it is either inaccessible or the hospital is overcrowded, they might choose to hide the sick family member, and Pasung might be one of the few options available to the family.

#### Actors

The Ministry of Health, through the Directorate of Mental Health, oversaw the majority of the policies and the psychiatric institution served as a frontline at the local and state level. In this Era, the community (i.e., the family) was partially engaged with the responsibility of guarding persons with mental ill-health while the person themself lost their autonomy to make decisions about their medical treatment. Yet the Ministry of Law and Human Rights established rules outside of the health system, such as penal codes, that constrained how a family should care for an ill member of their family. So, with the dominance of the biomedical system on the one hand and the penal code on the other, lack of access to mental health support and lack of community support continued to leave families with limited care options.

#### Process

During the First Era, Indonesia was a legally established country. As previously stated, much of the policy was passed down from the Dutch, including the Indonesian penal codes, which were taken from the Wetboek van Strafrecht (WvS) and was enacted by the Penal Codes Legislation No.1 of 1946 [39]. It was reasonable that much of the policy-making process was top-down during this time period. This circumstance was exacerbated by war, turmoil, and the economic crisis [40–42].

**Fig. 1:**
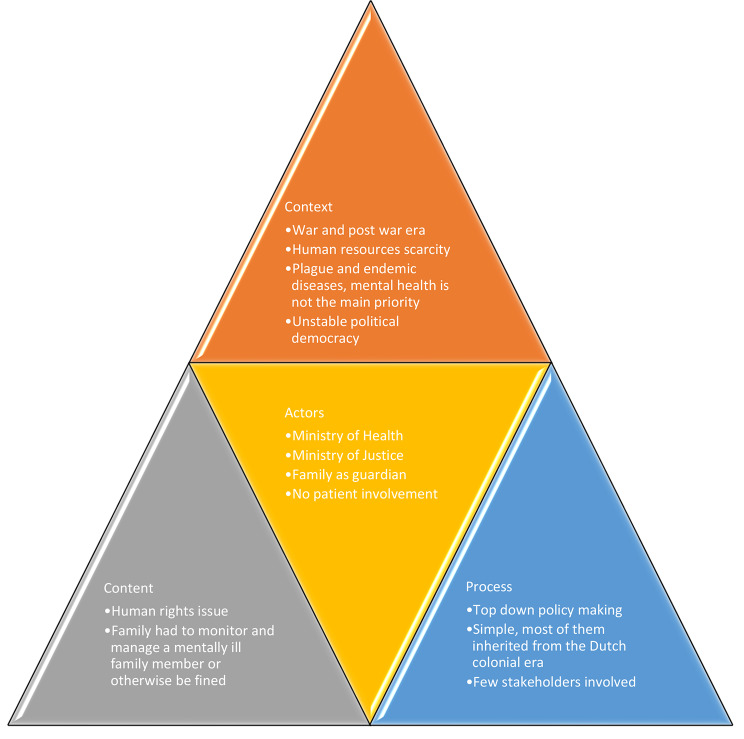
Health Policy Analysis on Pasung in the First Era of Indonesia 1945–1965

In terms of health policy, the Minister of Health develops legislation and policies at the national level in conjunction with a variety of stakeholders. The responsibility for producing implementation plans with specified targets, indicators, funds, and timetables rests to the provincial departments of health. Provincial agencies are also in charge of monitoring and evaluating national policy and legislation that has been implemented. Provincial districts (divisions of provinces) are in charge of implementing interventions on a local level in accordance with national and provincial priorities [38]. The context, content, actors and process elements within the First Era are summarised in Fig. 1.

### The second Era/New Order era (1966–1998)

#### Context

The three approaches of prevention, treatment, and rehabilitation were proclaimed by the Directorate of Mental Health in 1966 as the foundation of a comprehensive mental health care system. At this time, the Indonesian government chose a paradigm similar to that of the previous Era, in which the major players were the psychiatric institutions. It was demonstrated by the establishment of an even greater number of psychiatric institutions in Indonesia, in addition to the four psychiatric hospitals (Bogor, Grogol, Lawang and Magelang) inherited from the Dutch, as part of their colonial rule in Indonesia. The number of patients across these hospitals was doubled in 1970, and a range of therapeutic approaches were introduced [31, 36, 43].

#### Content

Three primary articulations ruled mental health and specific policy to prohibit isolation and restriction throughout the Second Era. Contrary to popular belief during this period, which viewed mental illness as hazardous and requiring constant monitoring, the government supplied a more comprehensive approach during this time, although only inside the confines of the institutions.

Three policies relating to this Era are detailed below:

1. **Mental Health Act No.3 of 1966**
  The first Mental Health Act for Indonesia was issued in 1966 [44] at the beginning of what is known as the New Order Era. This first Mental Health Act was a brief regulation and consisted of only seven Chapters and 14 Articles. The government began to see mental health as a problem for national development, particularly health development. The Act influenced the policy agenda at the time to view mental health through a predominantly biomedical lens, as mentioned in the first Article where the definition of mental health was linked with the definition of mental health in medical science:
  “Mental health according to the current understanding of medical science is a condition that allows a person acquire an optimal physical, intellectual and emotional development and that development goes in harmony with the situation of other people”.
  Furthermore, in Article 1 subparagraph (b), mental illness as defined in subparagraph (a), is a disturbance in mental function that leads to mental health issues. The regulation mentioned neither Pasung nor restraint and seclusion. However, it regulated the circumstances of a person who can be admitted to a hospital. In Article 6, the regulation mentioned that admission to a hospital without the patient’s consent is against the law:
  If a patient is required to be treated in hospital, then from a legal point of view the right to freedom of movement of the patient is limited. As such that action can be categorized as criminal conduct unless the restriction on freedom of movement is based on a law
  Despite the legislation urging persons to respect the human rights of a person in terms of freedom of movement (Article 6), other Articles within the Act seem not to do so. For example, Article 5 mentioned that coercion is in place where the doctor who treats the person has the authority to send the person to hospital without their agreement. To make it a legal action, consent could be obtained from one of the following: the patient if she/he is deemed to have enough capacity to give consent; or, where this is not the case, the parent, spouse, or the guardian of the patient. In the case of emergency or disturbing the peace and public security, the police or the judge as specified in Article 5 can refer a patient to the hospital. The word “disturbing public security” is not defined clearly in Article 5; therefore, this Article might be used to commit someone to a mental health facility without their consent. For the sake of safety, a family or community may consign someone who is regarded as mentally ill to a mental institution and prohibit them from returning to the community.
  The first Mental Health Act for Indonesia was issued in 1966 [44] at the beginning of what is known as the New Order Era. This first Mental Health Act was a brief regulation and consisted of only seven Chapters and 14 Articles. The government began to see mental health as a problem for national development, particularly health development. The Act influenced the policy agenda at the time to view mental health through a predominantly biomedical lens, as mentioned in the first Article where the definition of mental health was linked with the definition of mental health in medical science:
  “Mental health according to the current understanding of medical science is a condition that allows a person acquire an optimal physical, intellectual and emotional development and that development goes in harmony with the situation of other people”.
  Furthermore, in Article 1 subparagraph (b), mental illness as defined in subparagraph (a), is a disturbance in mental function that leads to mental health issues. The regulation mentioned neither Pasung nor restraint and seclusion. However, it regulated the circumstances of a person who can be admitted to a hospital. In Article 6, the regulation mentioned that admission to a hospital without the patient’s consent is against the law:
  If a patient is required to be treated in hospital, then from a legal point of view the right to freedom of movement of the patient is limited. As such that action can be categorized as criminal conduct unless the restriction on freedom of movement is based on a law
  Despite the legislation urging persons to respect the human rights of a person in terms of freedom of movement (Article 6), other Articles within the Act seem not to do so. For example, Article 5 mentioned that coercion is in place where the doctor who treats the person has the authority to send the person to hospital without their agreement. To make it a legal action, consent could be obtained from one of the following: the patient if she/he is deemed to have enough capacity to give consent; or, where this is not the case, the parent, spouse, or the guardian of the patient. In the case of emergency or disturbing the peace and public security, the police or the judge as specified in Article 5 can refer a patient to the hospital. The word “disturbing public security” is not defined clearly in Article 5; therefore, this Article might be used to commit someone to a mental health facility without their consent. For the sake of safety, a family or community may consign someone who is regarded as mentally ill to a mental institution and prohibit them from returning to the community.
2. **Home Affairs Ministerial Decree PEM.29/6/15 on 11 November 1977**
  This Home Affairs ministerial directive [13], written to the Governors in all provincial levels throughout Indonesia, asks the public not to shackle persons with mental illnesses and to create public awareness about the importance of providing care for patients in psychiatric hospitals. The letter also includes directions for sub-district and village chiefs to take proactive measures to deal with patients in their communities. Despite the fact that the policy was only at the ministerial level, the government, which was governed by authoritarians, had a great desire to implement it. At the time, the majority of governors were army major generals. This is why the Ministry of Home Affairs took the lead in the Pasung movement rather than other ministries [45].
3. **Law on Health No 23 of 1992**
  In the Law on Health policy issued in 1992 [46], there are no specific Articles or verses that mention Pasung. Four Articles covered the issue of mental health and what the government responsibility for each community should do to prevent, promote and treat mental illness. In this policy, governments are beginning to change the ethos of mental health by emphasizing the importance of community to support the overall health of its members. In addition, the definition of mental health which covered the social and productivity aspects showed the commitment to tackle the links between poverty, being without a job and mental health (see Table 2, Article 1).
  Moreover, in this policy, the government tried to implement mental health services in an integrative way, as seen in Articles 10, 24 and 25. Despite the boundaries and definition of prevention and promotion in this policy being vague in relation to cure and treatment of mental illness, policy makers attempted to pave the way for identification of the need for mental health services to be implemented comprehensively (see Article 24 verse 2).

**Table 2:** Chapters and Articles Relevant to Mental Health in Law on Health No.23 of 1992

Chapter	Article
1: General Terms	Article 1 (1). Health is a state of complete physical, mental, and social well-being that enable a person to be socially and economically productive
VI: Health service	Article 10 To achieve an optimum health status for the community, health services are carried out with promotive, preventive, curative and rehabilitative which are carried out in a comprehensive, integrated, and sustainable manner. Article 11 (1 F). Health services as mentioned in verses 10 including mental health
Chapter VII Mental Health	Article 24 (1) Mental health is implemented to obtain an optimum of both intellectually and emotionally. (2) Mental health includes the promotion and prevention of mental health, prevention and treatment of psychosocial problems and mental illness, curative and recovery of people with mental illness. (3) Mental health is implemented at all levels by individuals, the family, school, work, community, supported by mental health service facilities and other facilities.
	Article 25 (1) The government shall provide treatment and care, recovery, and to people with mental disorder after the hospital treatment and or treatment into the community. (2) The government initiates, assists, and fosters community activities in the prevention and management of psychosocial problems and mental illness, treatment and care, recovery and shelter of former people with mental illness into the community.
	Article 26 (1) Person with mental illness who possibly disturbed the public order and security must be treated and cared for in mental health service facilities or other health service facilities. (2) Treatment and care for people with mental illness can be carried out at the request of the husband or wife or guardian or family members of the patient or at the initiative of the official responsible for security and order in the local area or a court judge if in a case there is a suspicion that the person concerned is a person with a mental disorder. (2) The government is responsible for establishing and operating a national education system that is governed by law.

Despite many improvements in mental health policy in general, this policy remained unchanged in nature, similar to the Penal Code [39] and Mental Health Act of 1966 [44], where coercion is used in situations where a doctor or authority determines that someone needs to be hospitalised (see Table 2 Article 26 verses 1 and 2). Psychiatrists (doctors) were given significant power by mental health legislation, which gave them the right and responsibility to hold patients and force them to take medication or undergo other therapy.

#### Actors

Similar to earlier Eras, the Ministry of Health controlled the bulk of policies through the Directorate of Mental Health (previously Department of Mental Health) despite the leading organization to combat Pasung being the Ministry of Home Affairs. Outpatient psychiatry clinics integrated in referral general hospitals were more common than psychiatric institutions during this Era. A number of treatments approaches, including prevention, treatment, and rehabilitation, were also implemented as the cornerstone of a comprehensive mental health care system [31]. To overcome Pasung, the government attempted to enlist the help of other stakeholders, such as the Ministry of Home Affairs. However, there has been only partial implementation of the policy, with the family, and local schools, workplaces and communities largely taking up this burden with the help of mental health professionals and other organisations. For example, unlike physical health, mental health was not taught in schools. While the family’s obligation to protect mentally ill individuals continued, the patient’s autonomy to make decisions about their medical treatment without being affected by both their health care provider and carer was minimal.

#### Process

**Fig. 2:**
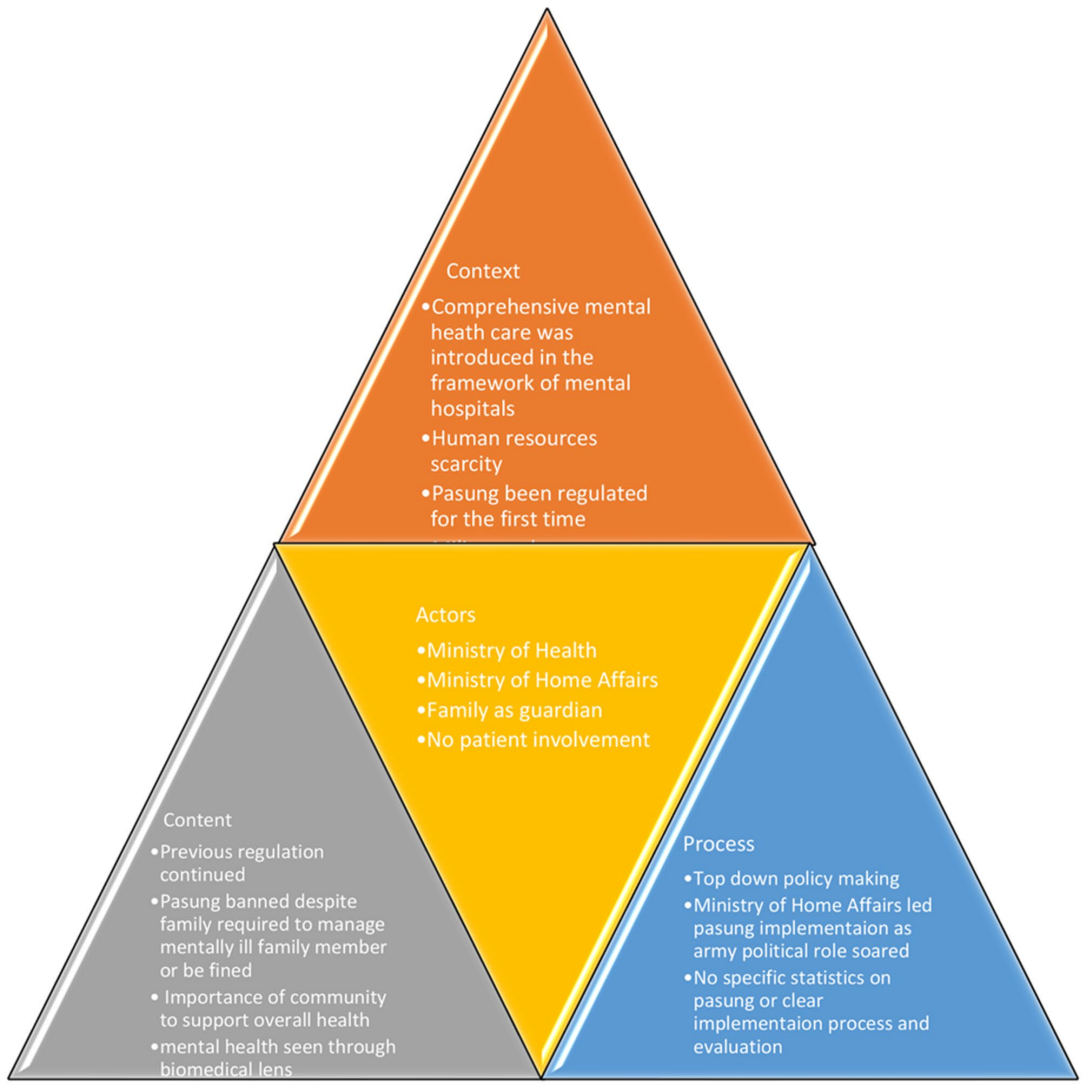
Health Policy Analysis on Pasung in the Second Era of Indonesia 1966–1998

Indonesia’s internal and international policies, including health and mental health, remained dominated by economic nationalism throughout the Suharto administration. The nationalist agenda had many forms and manifestations, but the essence remained the same as the previous Era [42]. Since the New Order’s inception in 1966, military ideology has emphasised the pursuit of economic development as a means of rescuing the country from the politico-economic disasters that occurred under the Old Era. From spoiler to essential supporter, the military adjusted its role. The military’s main role as a key supporter was to provide advice and policy recommendations to the president, as well as to criticise government [45, 47]. Most institutions, it should be noted, adopt top-down team management. This is how any ministerial and provincial level with an executive ladder is set up. It dismisses the involvement of civilians [34, 45]. The context, content, actors and process elements within the Second Era are summarised in Fig. 2.

### The third Era/Reformation era (1999-present)

#### Context

In the early Reformation Era, the resistance and students’ movement arose from the so-called urban middle class, resulting in a new civil movement. This had never been seen before; the government had previously faced few substantial challenges from either the military or civil society [30, 45, 48].

The health sector is one of the most important government initiatives in this Era. The government has enacted health-related legislation, including Health Law Number 36 of 2009. Then it was followed by the enactment of Law No.18 of 2014, which dealt with mental health. With the passage of the Law on Indonesia Social Security Scheme No 40 and the Law on Social Security Agency No 24, the transition from voluntary to mandatory social schemes began to support funding for health and mental health. Internationally, there was a global policy that shifted MDGs (2000–2015) to SDGs (2015–2030). In the MDGs, mental health was not specifically mentioned, but in SDGs, there are some articles (goals) that specifically mention mental health. Furthermore, during this Era, the care offered by dukuns, or indigenous healers, has also been given special consideration. Traditional medicine, according to Article 1 number 16 of the Health Law, is the therapy and/or treatment with techniques and substances that relate to empirically inherited experience and abilities that can be accounted for and implemented in line with societal standards. Moreover, pharmaceutical preparations in the form of traditional medicines and cosmetics, as well as medical equipment, must comply with the norms and/or requirements set out in Article 105 of the Health Law.

1. **Content**: In comparison to the previous Era, the government issued four times as many policies during this Era in terms of health and disability, which have included a broader definition that includes mental illness, mental health, and particular policy to prevent isolation and confinement throughout the third period. The following are the policies that were put in place during the time.
**The Amended 1945 Constitution (Amendments)**
  The 1945 Constitution of the Republic of Indonesia [38] has been amended four times during the Reformation Era, most notably in 1998 [49] with the insertion of the Human Rights Article. It now contains a dedicated chapter solely for human rights in Chapter XA. Three verses (i.e., Articles 28B, 28G and 28I) underline the protection against violence and torture or other treatment that degrades human dignity. As example, Article 28G states:
  Every person has the right to be free of torture and other degrading treatment that degrades human dignity.
2. **Human Rights Act Number 39 of 1999**
  The fulfilment of the right to health for persons with a mental disorder links with Indonesia’s human rights obligations. The Human Rights Act of 1999 [50] was issued long before Indonesia ratified the Convention on the Rights of Persons with Disabilities (CRPD) [51] in 2011 (see the law on CRPD No.19 2011). In Chapter II of this Act, the basic principle of Article 4 verse 1 mentions that every person has the right to life, the right not to be tortured, the right to personal freedom, thoughts and conscience, the right to religion, the right not to be enslaved, the right to be recognized as a person and equal before the law, and the right not to be prosecuted on the basis of retroactive law are human rights that cannot be reduced under any circumstances and by anyone.
  Furthermore, Article 9 states that: (1) Everyone has the right to live, maintain life and improve their standard of living; (2) Everyone has the right to live in peace, security, happiness, physical and spiritual prosperity; and (3) Everyone has the right to a great and healthy living environment. By issuing this legislation, the government wished to emphasize that torture, including seclusion and restraint of people suspected of having mental illness, is an act that is contrary to human rights. The use of seclusion or physical restriction as a technique for a hospital’s administrative convenience or inpatient ward management is regarded as a violation of human rights. The Article stressed that staff can only administer seclusion for brief periods of time as a method of crisis management or when it is the only way to prevent urgent or impending harm to the patient or others.
  During this time, there were no regulations in place that expressly addressed people with disabilities. Existing regulations are dispersed and cover areas such as education, health, accessibility, and employment that affect people with disabilities. It was also at this time that new phrases were coined, and first applied to people with impairments. ‘People’s disability’ and ‘handicap’ are two examples of these phrases, and they apply to a variety of disabilities (blind, quadriplegic, and impaired speech). At the end of the Era, the term “persons with disabilities” has emerged in the regulations.
3. **Law on Indonesia Social Security Scheme No. 40**
  The Law on Indonesia Social Security Scheme No. 40 [52] is a game-changing step towards meeting the Indonesian government’s goal of Universal Health Coverage (UHC). Articles 3 of this regulation stated that “Social security is a form of social protection to ensure that all people can meet their basic needs for a decent life”. The Indonesian social security programme is currently undergoing a fundamental overhaul in order to improve the existing system’s performance for beneficiaries and to expand social security coverage to more people, including those with psychosocial disabilities. The Indonesian government paid the insurance premiums for those in need and those with psychosocial disabilities, as stated in articles 20 - “Participants in health insurance are anyone who has paid contributions or has had contributions paid for them by the government.” Furthermore, Article 21 (3) of this Law states, “Participants who have permanent total disability and are unable to pay their contributions are reimbursed by the government.”
  By the enactment of this Law, as stated in Article 22, the participant could get services that include promotive, preventive, curative and rehabilitative services, including medicines and consumable medical materials. This regulation, as stated in Article 23, also strives for people to seek treatment at the nearest health facilities as all community centres and hospital cooperate to this system.
4. **Law on Health No 36 of 2009**
  The Law on Health policy issued in 2009 [53] has similar issues of concern with the previous Law on Health policy issued in 1992 in term of Pasung regulation, where we found no information directly regarding Pasung. The content regarding mental health in chapter IX consisted of 9 Articles (see Table 3). The difference between the previous and the 2009 Law in Health policy is described in several Articles below.
  In Article 144 (3) and Article 147 verse 1, the responsibility is stated not only of the government but also local government and community to hold shared responsibility for implementing the mental health service. This version was issued after the Reformation Era and the Decentralization Era where all of the Indonesian provinces and districts have their own autonomy in terms of managing funding and local resources and the central government manages the overarching policy and some of the resources. Moreover, community involvement was introduced to increase the coverage of mental health services. The government moved its thinking to be more widely open to the community being involved in the mental health service such as mental health at work and mental health at school initiatives.
  Article 146 described how education and information regarding mental health is a must to protect the human rights of people with mental illness. Due to stigma, discrimination, and a lack of legal protection, individuals are exposed to human rights breaches in the community and in a range of services. Improved media reporting and public education were deemed as useful strategies to reduce human rights concerns.
  Article146 (2) is another example of where human rights issue has been included in this policy document, as exemplified in verse (1) which aimed at avoiding violations of the human rights of a person who is considered to have a mental health disorder. Article 148 verse 1 further stated that people with mental illness have equal rights to other citizens.
  The minimum standard requirement for mental health facilities started to be regulated in this law, which mentioned in Article 147 (3) that specific health service facilities demonstrate standard requirements that are in accordance with those mentioned as needed in the regulation to effectively treat and care for people with mental health illness. During this Third Era of reform, policies focusing on disadvantage and social exclusion have emerged from the new determination to address the links between poverty, unemployment, and mental illness. This was covered in Article 149 (1).
  Despite many improvements in this policy, psychiatry continues to separate mental illness from the person’s broader social and environmental context and surroundings. Mental health issues and psychosocial problems are both defined as abnormal personal experiences. Social and cultural variables are secondary at best and may or may not be considered (see Article 1 definition of health). This is partly due to the fact that the majority of psychiatric contacts take place in hospitals and clinics where the treatment focus is only on the person‘s clinical symptoms, whether through medications or psychotherapy (see Article 147 (2–3), Article 149 (1,2,3) and Article 150 (1)). (See Table 3).
5. **‘Towards Indonesia Free of Pasung’ 2010**
  With the increasing number of people in Pasung, the Ministry of Health launched the campaign of ‘Toward Indonesia Free from Pasung’ or Indonesia Bebas Pasung in October 2010 [14]. Apart from the increasing incidence of Pasung, this campaign is based on increasing advocacy and encouragement from many organizations including human rights organizations, mental health organizations, and extensive media information. The regulations used as the basis of the campaign are the Mental Health Act of 1966 [44], the 1992 Health Law [46] and the Letter of the Ministry of Home Affairs 1977 [13], which ordered the public not to implement shackles for people with mental health issues and to raise public awareness to hand over care for these individuals to mental hospitals or other mental health facilities. The letter also contains instructions for all governors, mayors, district and village leaders to actively take initiatives and steps in dealing with people in Pasung in their area.
  The Ministry of Health added that to meet the needs of people with mental health issues who are confined and neglected, comprehensive efforts are needed from all aspects: health, economic, and social. This effort regulates the role of government, local government and the community. The Ministry of Health further stressed that the central government and local (provincial and district) governments are responsible for the equitable distribution of mental health service facilities by involving the participation of the community, including the financing of treatment and care for people with mental health issues for the poor. The government and local governments not only find cases of Pasung and then release them, but also provide education to the public to discourage them from using Pasung.
  Community health centers are empowered so that they can become the first place for contact and delivery of mental health services and that they must also provide the necessary treatment. General Hospitals must provide beds so that they can treat people with mental health issues that require treatment. Psychiatric hospitals, apart from being a referral center, must also be able to become a center for mental health development for health services in their area. Community participation is expected to enable identification of individuals with mental health issues in the community, avoid shackles and encourage community members to seek treatment and carry out control. The program also targets the decision-makers, non-governmental organizations, professional organizations, community leaders, health leaders, special groups, related sectors at the central and regional levels and individuals who experience chronic diseases as well as people with mental health issues [50].
6. **Law on Social Security Agency No. 24 of 2011**
  To achieve the goals outlined in Law No. 40 of 2004, the government established an administrative body in the form of a legal entity based on the principles of mutual cooperation, non-profit, openness, prudence, accountability, portability, mandatory participation, trust funds, and the results of managing social security funds entirely for programme development and the best interests of participants. In Article 2 Law No. 24 of 2011 [54], this social security agency was divided into two parts: the health agency and the workers agency. The formation of this social agency body actually was slightly longer than expected as mandated in Article 52 of Law No.40 of 2004 which stated, “All provisions governing the Social Security Administering Body as referred to in paragraph (1) are adjusted to this Law no later than 5 (five) years after this Law is promulgated.” It took seven years to establish the administering body.
  In relation with Pasung, none of the Articles mentioned directly either Pasung or mental illness; but, in the Article 19 of this law, the government emphasized that the insurance premium for those who are in need will be paid by the government. Indonesians whose income falls below the poverty line will be considered low-income earners and will thus be eligible for government assistance. The law itself does not regulate how the insurance premium will be paid and does not mention what categories for psychosocial disabilities are covered by the law. The government’s plan to subsidise coverage for people on low-incomes with psychosocial problems is questionable given the fact that the majority of persons in Pasung are on low incomes and uninsured [55].
7. **Mental Health Act No 18 of 2014**
  The Mental Health Act No. 18 of 2014 [15] has several progressive scopes of norms, such as focusing on ‘People with Mental Health Problems’ (Orang Dengan Masalah Kejiwaan), who are individuals who are at risk of developing mental illness, and ‘People with Mental illness’ (Orang Dengan Gangguan Jiwa), who are individuals who have been diagnosed with a mental illness (see Article 2b, Table 4), as well as treatment and care approaches. Despite the existence of the regulation for individuals with mental health issues, the policy is still not regarded by the community as the best option for them due to the persistence of Pasung and mental health problems.
  We have highlighted detail within Article 86 (see Table 4) that explicitly states that perpetrators of Pasung may be subject to sanctions or punishments. Despite vague messages from this policy regarding what kind of criminal punishment will be implemented, the policy strongly suggests that perpetrators of Pasung are to be treated similarly to those who commit other criminal acts, though most of them are the person’s families and the closest neighbour.
8. ** Law No.19 of 2016 concerning the rights of persons with disabilities**
  The CRPD was ratified by Indonesia and incorporated into Law No.19 of 2011 [51] concerning the Rights of Persons with Disabilities, which was then followed by Law No.8 of 2016 [56] which replaced the previous law. Persons who are referred to as ‘Persons with Disabilities’ in this new law context as mentioned in Article 1 and Article 4 include those who have long-term physical, mental, intellectual, or sensory disabilities that can hamper their full and effective involvement in a society based on equality when confronted with numerous difficulties.
  The 2016 Act explains the rights of persons with disabilities as specified in Article 5 verse 1: “Every person with disabilities must be free from torture or cruel, inhuman, degrading treatment of human dignity, free from exploitation, violence and arbitrary treatment, and has the right to respect for mental integrity and physically based on similarities with other people.”
  Another important point is stigma, as cited in Article 7, is that persons with disabilities have the right to be stigma-free, which encompasses freedom from harassment, humiliation, and negative labelling related to their disability condition. The enactment of the law demonstrates the Government of Indonesia’s commitment and seriousness in respecting, protecting, and fulfilling the rights of persons with disabilities, which are intended to promote their well-being, including the right to protection and social services in the context of independence, as well as in an emergency.
9. ** Ministerial Decree No. 36 of 2016 Healthy Indonesia Program through Family Approach (Program Indonesia Sehat dengan Pendekatan Keluarga – PIS-four PK)**
  The Healthy Indonesia Program through Family Approach (Program Indonesia Sehat dengan Pendekatan Keluarga) is a strategy implemented by the Community Health Centre using a family approach (Puskesmas) [57]. PIS-four PK’s priority areas, as mentioned in Article 2, are reducing maternal and infant mortality, controlling the prevalence of stunting in children, controlling infectious diseases, and controlling noncommunicable diseases, particularly hypertension, diabetes mellitus, and mental disorders. Primary Health Centres should visit families in their coverage area as part of this programme to assess 12 health indicators in each family as mentioned in Article 3, one of which is how family members with mental illnesses are treated. People suffering from mental illnesses (including Pasung cases) would then be identified and treated.
10. ** Stop Pasung Movement (Gerakan Stop Pemasungan/GSP)**
  The Free Pasung Program is presently led by the Ministry of Social Welfare, which launched “Gerakan Stop Pemasungan” in 2017, in collaboration with the Ministry of Health at the national level [58]. Different target dates are set and revised as both Ministries battle with the immensity of achieving a Pasung-free Indonesia. The Ministry of Social Welfare recently stated that Indonesia would be Pasung-free by the end of 2019, then the Ministry of Health suggested that this would not be achieved until 2023 [58].
  The transfer of the Free Pasung Program from the Ministry of Health to the Ministry of Social Affairs by the central government in 2017 changed the way the program was implemented. While the Ministry of Health developed a Free Pasung Program on a more institutional basis to care for those who have experienced Pasung, the Ministry of Social Affairs is establishing community-based pilot initiatives with a focus on social rehabilitation. The Ministry of Social Affairs is implementing recovery-oriented practices in accordance with their 2013 Social Rehabilitation Program Development Plan, which aims to enable people with mental illnesses who have been in Pasung or experienced homelessness to return to their families as participating and productive citizens while also providing accessible support services for people in their communities [58].
11. ** Ministerial Decree on Stop Pasung no 54 2017**
  Over the past decade, there has been a profound effort in Indonesian mental health policy to shift mental health services to the community [53, 58]. Despite implementation being far from successful, the spirit to deinstitutionalize the system is a key talking point among mental health service workers. Despite the changing role in the Ministry which led the Free Pasung movement, the Ministry of Health enacted the new policy regarding Pasung via its ministerial Decree No.54, 2017 [59]. This decree reinforced and elaborated on the blueprint, emphasising the link between the establishment of a comprehensive mental health system and the eradication of Pasung in Indonesia.
  This regulation depicts the Indonesian mental health service system, referencing other regulations such as Jaminan Kesehatan Nasional (Law No.40 of 2004 Concerning the Indonesia Social Security Scheme) [52] and Badan Penyelengara Jaminan Sosial (Law No.24 of 2011 concerning Social Security Agency) [54]. Primary care facilities, such as Puskesmas (community health services), Sub Health Centres, Polyclinics, Indonesian Armed Forces, and family doctors using outpatients with funds granted by the legal provisions, are the primary providers of care under this system. Severe cases should be sent to a district hospital, a private hospital, or a mental institution. Other treatment options include going straight from the public to the public or a psychiatric facility. Following treatment, the psychiatric patient must return to the community via a return referral to a primary health clinic where they will continue outpatient treatment. Even though it appears to be a faultless system, many services such as housing and rehabilitation services to reintegrate patients into society, as well as outreach programs such as home visits by Puskesmas employees and cadres, are not fully covered by these insurance systems.
  As part of the decentralization process, the legislation also encourages local governments to give funds for treatment as mentioned in Article 3. Although decentralization has positive intentions in terms of equal distribution of mental health care, it has detrimental consequences that have resulted in service fragmentation, which has been matched by rising fragmentation of management and funding. The burgeoning mental health system lacked a focal point of planning and responsibility to exert the type of stewardship historically done by state mental health authorities. In Indonesia, health and disability insurance schemes for mental health treatments in the community have yet to commence. Another major concern is how they would be compensated under this plan.
12. ** Ministry of Health Ministerial Decree on Minimum Services Standard on Health No 4**
  As stated in Article 1 of this Ministerial Decree, the district government is responsible for providing appropriate care to all people suffering from mental illnesses. Furthermore, the district and province are required to implement the 12 standards of the Healthy Indonesia Program through Family Approach, as stated in Article 2 point 3, of which mental health is one of the 12 indicators. Article 2 point 1 also allows districts and provinces to include another indicator relevant to the needs of their district. Besides that, as stated in Article 2 point 3, the promotion of mental health has been a priority and is situated within the larger field of health promotion, alongside mental illness prevention and treatment and rehabilitation for people with mental illnesses and disabilities. Community involvement has also been regarded as an important part in this law where Article 2 point 6 stated that trained health cadres are to perform certain types of basic services outside of facility health services under the supervision of health personnel. The inclusion of the community is important in the continuation of the Free Pasung Program because community members are usually the first persons who discover cases of Pasung in the community [60].
13. ** West Java Provincial Regulation on Mental Health Service No.5 2018**
  While six provinces have gubernatorial regulations (Pergub) for mental health services (which is Aceh, East Java, Yogyakarta, West Nusa Tenggara, Bangka Belitung, and Central Java) [19], West Java Province claimed to be the first in Indonesia to issue the Regional Regulation for Mental Health (Perda) in 2018 at the provincial level to govern mental health services throughout the province. There are 13 chapters and 88 Articles in this Act [61] (see Table 5). The regional regulation is a derivative of the Mental Health Act No.18 of 2014 [15], which was enacted in response to specific conditions in the province. Many provisions in this regulation, such as the definition and management of Pasung, fully follow the Mental Health Act.

**Table 3:** Chapters and Articles Relevant to Mental Health in Law on Health no 36 2009

Chapter	Article
1: General Terms	Article 1 (1). Health is a state of complete physical, mental, and social well-being that enable a person to be socially and economically productive
IX: Mental Health	Article 144 (1) Mental health service is aimed to ensure every people have a healthy life, free from fear, stress, and other symptom related with mental disorder. (2) Mental health services as referred in verses (1) consists of preventive, promotive, curative, rehabilitation of patients with mental illness and psychosocial problems. (3) Mental health services as referred to in verses (1) is the joint responsibility of the Government, local government, and the community. (4) Government, local government, and community responsible for creating mental health service at the highest possible level and ensure the availability, accessibility, quality and equity of mental health services as referred to in verses (2). (5) The government and local governments are obliged to develop community based mental health service as part of integrated mental health service, including accessibility to a community for mental health services.
	Article 145 The government, local government and community ensure mental health service in all levels including preventive, promotive, curative and rehabilitative, also in the workplace as referred to in Article 144 verses (3).
	Article 146 (1) The public has the right to obtain faultless information and education regarding mental health. (2) The rights as referred to in verses (1) are aimed at avoiding violations of the human rights of a person who is considered to have a mental health disorder. (3) The government and local governments are obliged to provide information and education services on mental health.
	Article 147 1) The endeavour to cure people with mental illness are the responsibility of the Government, local governments and the community. (2) The healing process as referred to in verses (1) are carried out by authorized health personnel and in the proper place to respect the patient’s human rights. (3) A specific health service facilities with standard requirements and are in accordance as mentioned in the regulation is needed to treat care people with mental health illness
	Article 148 (1) People with mental illness have the equal rights as other citizens. (2) The rights as referred to in verses (1) include equal treatment in every aspect of life, unless the laws and regulations state otherwise.
	Article 149 (1) People with mental illness who are neglected, homeless, threaten the safety of himself and/or others, and/or disturb order and/or security the general public are required to receive treatment and care in health care facilities. (2) The central government, local government, and the community are obliged to give treatment and care at the mental health facilities for people with mental illness who are abandoned, homeless, threatening safety himself and/or others, and/or interfere public order and/or security. (3) The central government and local governments are responsible on equal distribution of mental health facilities by involving public actively. (4) The Government and local governments responsibility as referred to in verse (2) includes financing of the poor.
	Article 150 1) Mental health examination for the benefit of law enforcement (visum et repertum Psychiatricum) can only be done by psychiatrist at mental health facilities. (2) Determination of the legal competence status of a person who are suspected of having a mental disorder conducted by a doctor who have the expertise and competence in accordance with professional standards.
	Article 151 Further provisions regarding mental health service will be regulated with Government Regulation.

**Table 4:** Chapters and Articles Relevant to Mental Health in Law on Health no.14 2018

CHAPTER	CONTENT
Chapter 1: General Terms	Article 2b Mental health treatment principle should be based on humanity. “Humanity principle” mean that the implementation of Mental Health treatment for People with mental problems and mental illness is carried out humanely in accordance with human dignity. **For example, no restraints and so forth.**
Chapter V	Article 70 part 1 (1) People with mental illness (ODGJ) reserve the right to: a. Access mental health services b. Get mental health services in accordance with the predetermined standard. c. Get a guarantee for the availability of psychopharmaceutical drugs according to their needs. d. Have the right to give consent for the medical action taken against him. e. Obtain honest and complete information about their (people with mental health problems) mental health data including actions and treatments they have or will receive from health workers with competence in the field of Mental Health. f. **Get protection from every form of neglect, violence, exploitation, and discrimination.** g. get social needs according to the level of mental illness; and h. Manage their own assets and/or those handed over to them.
Chapter IX: Criminal Provisions	**Article 86** **Anyone who intentionally detains, neglects, abuses and/or induces other people to carry out shackles, neglect, and/or violence against People with Mental Problems (ODMK) and People with mental illness (ODGJ) or other actions that violate the human rights of People with Mental Problems (ODMK) and People with mental illness (ODGJ), shall be punished in accordance with statutory provisions.**

**Table 5:** Chapters and Articles Relevant to Pasung in West Java Provincial Regulation on Mental Health Service No.5 2018

CHAPTER	CONTENT
Chapter 1: General Terms	Article 1 verses 13 **Pasung, as described in this local legislation, might include different forms of mechanical or non-mechanical confinement that isolates individuals from the community, as well as other types of coercion, which including making it difficult for them to obtain health care.**
Chapter 2 Mental Health service	Article 4 Preventive, promotive, curative, and rehabilitative mental health services are provided in a comprehensive, integrated, and long-term manner.
	Article 5 This provision serves as a model for all districts and cities in developing policies and implementing mental health services.
	Article 10 **Mental health program is carried out by reducing stigma, myths, discrimination, violations of human rights for People with Mental Problems (ODMK), who are individuals who are at risk of developing mental illness, and People with Mental illness (ODGJ), and treating them as part of the family and community.**
Chapter V	Article 20.3 People with mental illnesses who endanger their own or others’ safety, or who violate public order and security, are obligated to seek treatment and care in a health care facility. Security personnel might be ordered by health workers, and emergency treatment could be administered if needed. Article 20.2 Consent for the in-hospitalization of an aggressive patient might be acquired from the patient’s spouse, parent, children, or other relative above the age of 17, or an authorised authority as defined by this legislation.
Chapter VI Organisation	**Article 72** **The provincial government and district/city governments manage street psychotics or psychotic homeless people and shackle victims through a rapid response team that includes at least elements of the regional apparatus that manage and control affairs in the fields of health, social, population, and civil records, manpower, and maintenance of public and community peace, non-governmental organisations, and other related elements.**

The Current data on the number of Pasung instances or regions declared free from Pasung, is speculative. Every analysis must begin with a locally grounded case study because there is no reliable centralised data monitoring method for tracking Free Pasung Program development. Hence, in this policy analysis, we also integrated the West Java local context to measure the implementation of the policy at grass root level.

Although West Java has many of the building blocks outlined in the 2017 national rule, such as some primary, secondary, tertiary, and outreach care, there are many gaps in the delivery of essential services. Civil society and other unconnected government activities cover some of these gaps. The presence of community health advocates, for example, despite the fact that some of them had not gotten the required mental health training and were only dealing with neonates, mothers, and the elderly in minor situations. The West Java health district stated that primary care mental health programs were operational but in fact the ministry of health stated contradictory which only 20% of this primary health care provide mental health service. The most accessible primary care clinics, on the other hand, lacked mental health expertise; Puskesmas mental health nurses performed the best they could with limited resources and training, including limited medications and referral routes that were plagued by access concerns. Because of their remoteness, limited hours of operation, and absence of emergency services, psychiatric and psychological clinics were inaccessible. A tertiary hospital was dispatched to the bulk of serious and emergency patients.

Despite West Java being the first province to pass provincial mental health legislation, some of the provisions of the regulation cannot be applied immediately since they require the gubernatorial (regulation at the provincial level) regulation indicated in Articles 86 and 87. For example, it is still unclear how stakeholder cooperation or district-provincial cooperation works.

#### Actors

During this time, the Ministry of Health did not act alone; many other stakeholders were involved, including the Ministry of Law and Human Rights, which was in charge of regulating and enforcing human rights, the Ministry of Social Affairs, which was responsible for administering and enforcing Disability Rights, and the Ministry of Finance, which was in charge of funding the mental health service sector. The National Commission on Human Rights, on the other hand, has an independent task of investigating complaints about discrimination and human rights violations against people with mental illnesses, as well as launching an advocacy campaign to protect the rights of people with mental illnesses. Psychiatric hospitals, particularly those owned by the government, do not serve as a baseline for mental health care; rather, public hospitals are mandated to provide mental health care. Community health centres are also responsible with delivering primary care services, including early detection and treatment of acute conditions. The public is encouraged to get involved, whether through mental health groups, volunteering, or serving as drug compliance supervisors. With the support of mental health services and other institutions, the family, school, workplace, and community take on this burden. While the family’s responsibility to safeguard people with mental health issues remains, the person’s autonomy to make medical decisions without being influenced by both their health care professional and their caregiver is fading.

#### Process

There have undoubtedly been some improvements in particular areas and with respect to specific challenges related to Pasung during this Third Era. In comparison to the previous period, mental health problems have received more government attention in terms of policy. Despite the fact that the budget is shrinking, consumer organisations have emerged that now cover practically all mental health issues [31, 37, 62]. The Indonesian Schizophrenia Support Community (Komunitas Peduli Skizofrenia Indonesia/KPSI) and Bipolar Care Indonesia (BCI) are two such organisations that address specific illnesses like schizophrenia and bipolar disorder. These organisations play a critical role in raising public awareness about mental health issues. They also advocate for better mental health legislation and improvements in the mental health treatment system [62].

**Fig. 3:**
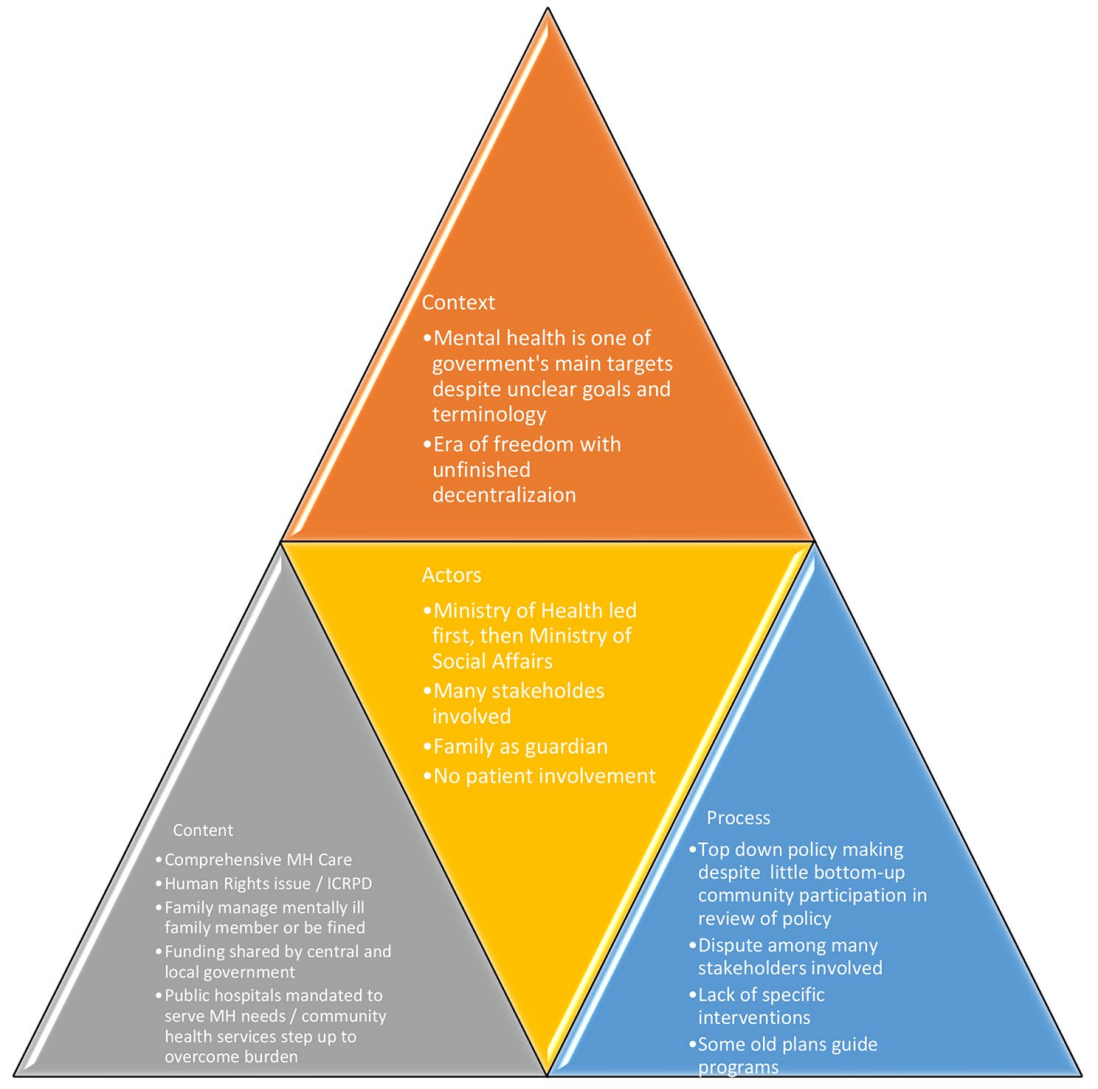
Health Policy Analysis on Pasung in the Third Era of Indonesia 1999-present

The Indonesian government is now paying greater attention to mental health issues than it was previously; many ministries are involved. Indonesia Bebas Pasung is a priority for the Ministry of Social Welfare. A special report on mental health issues was produced by the Ministry of Law and Human Rights, which urged that the government to take the lead in reforming the mental health care system. Mental health issues in the workplace are receiving attention from the Ministry of Manpower and, as stated above, the National Commission on Human Rights has launched an advocacy campaign to protect the rights of people with mental illnesses. All of these events aided in the passage of several policies on Pasung, such as the Free Pasung Program and the Mental Health Act of 2014. The context, content, actors and process elements within the Third Era are summarised in Fig. 3.

## Discussion

The objective of this policy analysis is to examine Pasung policy in Indonesia. It focuses on four essential aspects: the policy’s content, the people involved in policy change, the methods for designing and implementing change, and the policy’s context. In Indonesia’s First Era, neither Pasung nor mental health policies were implemented in the nation’s mental health system. This was due to a combination of causes, including war [34, 35], the scarcity of human resources, and the revival of both endemic and epidemic disease [34]. Furthermore, the Penal Code [39] enacted a year following the proclamation of Indonesia in 1946 requiring the family to keep the person with mental illness at home or accompany them at all times, introduced another layer of difficulty, since the family is obliged to take on these responsibilities. As a result, many people experiencing mental illnesses were neglected and denied medical treatment and imprisoned in their own community [31].

The Ministry of Health reformed mental health treatment from American standards during the Second Era, resulting in the construction of numerous transit homes, agricultural settlements, and a separate psychiatric institution, notably in Java [31]. However, the medicalization of mental health issues tends to ignore the person’s subjective experience of the condition in favour of delivering accessible mental health care within a complicated health system and biomedical paradigm (see Mental Health Act 1966 Article 1). Due to the country’s inadequate health services, people in remote locations still have limited access to care [36, 37].

Pasung policy was first introduced in 1977 [13] in the legislation in the form of a Ministerial Letter from the Department of Home Affairs sent to all governors, urging them to stop using Pasung and instead deliver their mentally ill to a psychiatric hospital. This letter was in accordance with Article 6 Mental Health Act 3 of 1966 [44] which legislated that people with mental illnesses should be treated and medicated in a treatment facility. The increase in the number of people attending psychiatric institutions represented local society’s greater participation in this method of treatment. However, this resulted in hospitals becoming overcrowded, prompting many families to keep their family at home. Significant human rights violations have occurred as a result of this situation, with people with serious mental illness and people with psychosocial disabilities subject to Pasung, arbitrary and prolonged hospital detention, and involuntary treatment both in the community and in hospitals [36, 37, 64]. Despite the introduction of law in this period making it illegal to shackle a mentally ill individual, the practise persisted.

In the Third Era, the Indonesian government has become increasingly aware of the need to address mental health issues. The Indonesian government has enacted health-related legislation, such as Health Law Number 36 of 2009, the Free Pasung Program initiatives of 2010, and the Mental Health Act No.18 of 2014 which each purport to deal with mental health. Policies concentrating on disadvantage and social exclusion have come from a renewed resolve to address the links between poverty, unemployment, and mental illness during this period. For these issues, the government passed the Law on Indonesia Social Security Scheme No.40 of 2004 and the Law on Social Security Agency No.24 of 2011 which started the transition from voluntary to mandatory social schemes to support funding for health and mental health. This new insurance enabled people with mental illness to get treatment at mental hospitals or other health services. This new policy would directly affect the eradication of Pasung since financial problems are one of the greatest barriers to seeking and receiving mental health services. Internationally, there was also a global policy that shifted MDGs (2000–2015) to SDGs (2015–2030) which included mention of mental health [65].

People with mental health problems who are neglected, homeless, and considered at high risk for harming self or others are obligated to get treatment and care in health care institutions during this Era. In 2010, the Ministry of Health became the leader of the Free Pasung Program, which had previously been overseen by the Ministry of Home Affairs, three decades after the prohibition on Pasung was enacted. It was then followed by the enactment of The National Mental Health Act of 2014 [15] which reaffirms that any aggression against a person with a mental health condition, including Pasung, was deemed criminal. This Act also served as a foundation for the creation of a comprehensive mental health system centred mostly in institutions.

Despite this progress, mental health difficulties are inextricably tied to Indonesia’s difficulty in implementing health programs. Comprehensive mental health care, which includes prevention, curation, promotion, early intervention and rehabilitation, cannot be implemented unless a government regulation is adopted under this legislation. In addition, the Mental Health Act of 2014 [15] requires at least five government regulations, one presidential regulation, and three ministerial regulations to be properly implemented. These elements constitute a significant hurdle since developing the regulation is a time-consuming procedure that involves dealing with various parties’ interests.

The Ministry of Health then issued two new policies to implement comprehensive mental health service. First, in 2016, the “Healthy Indonesia Program through Family Approach” (Program Indonesia Sehat dengan Pendekatan Keluarga), then Regulation No.4 of 2019 on Minimum Services on Health. are seen as important tools to combat pasung, even though their success is not yet evaluated.

Furthermore, the Free Pasung Program was conducted without any detailed instructions until the Health Ministerial Decree on Stop Pasung No. 54 of 2017 [59], which was issued over seven years after the program started. In my experience, the lack of detailed guidance causes health workers at the grassroots to be perplexed about how to implement the Free Pasung Program. Due to the large number of stakeholders involved, each actor has a different interpretation of how to manage pasung depending on their organization’s interests. For example, a person in Pasung who has a physical disease that should be treated in a general hospital before being admitted to a psychiatric institution frequently goes untreated and is left in Pasung because no stakeholder can holistically manage these comorbid conditions.

Collaboration in healthcare is a multifaceted process that brings together two or more people, sometimes from different professional disciplines, to work towards common goals and objectives [66, 67]. Healthcare providers and patients alike benefit from interdisciplinary teamwork. The level of collaboration among providers can have a direct impact on patient outcomes [67, 68]. There are a number of reasons why this collaboration appears to be failing to eliminate Pasung. First and foremost are the job descriptions of all stakeholders. Another major element is that, since the introduction of the Free Pasung Program implementation in 2010, there has been no technical policy or instruction that formalises the role of each stakeholder.

This situation is exacerbated by an incomplete decentralisation of healthcare policymaking and service delivery, particularly at the primary level. Ideally, as mandated by Ministerial Decree No.4 of 2019, provinces and municipal governments develop their own plans and programmes to respond to specific local issues in accordance with national policy objectives, strategies, and priorities [69, 70]. In practise, as in many developing countries, local governments struggle with an unclear direction of change, as well as a dual dilemma of dealing with both pre-existing chronic problems that necessitate more resources [70, 71], and financing problems [70]. It has been identified that ongoing lack of awareness, low prioritisation, and lack of commitment by stakeholders are major impediments to the development of mental health services [21, 69–72].

It is also possible that policymakers have overlooked international obligations and lessons learned from successful policymaking in comparable regional countries, resulting in disparities in target-setting, implementation mechanisms, and evaluation. One successful example is China, which implemented the 686 programme to scale up nationwide basic mental health services with the goal of improving access to evidence-based care and promoting human rights for people with severe mental disorders. The program “unlocked” and provided continuous mental health care to people with severe mental disorders who were found in restraints and largely untreated in their family homes [73]. To date, that programme has developed an increasingly clear model of services that moves mental health care out of the institution-based mental hospital and into community settings, connecting provincial and district hospitals to primary level health clinics that provide community outreach services. Rather than relying on older models for providing mental health services in primary care settings, which rely on training primary care doctors and nurses to recognise and respond to a subset of people suffering from mental illnesses who appear in their clinics, as has been implemented in Indonesia, the programme building multifunctional teams and sending them into the community represents a greater commitment of resources. The program also shifts its paradigm from medication only into more comprehensive rehabilitative and preventive care at its core [73, 74].

Despite the Indonesian government claims that the Free Pasung Program had successfully reduced the number of Pasung cases from 18,880 to 2017 to 12,220 in 2018 [2, 17, 75], its repeated revisions of the program (2010–2017 and 2019) have cast doubt on the actual number of people still in Pasung. In addition, in 2013, the Indonesian National Health Surveys revealing that, of households identified as containing a person with mental ill-health, approximately 14.3% lived in Pasung [28]. In a comparable survey conducted in 2018, the percentage of households with persons who are predicted to have a mental illness increased from 1,7 per mile in 2013 to 7 per mile in 2018 and 14% household has Pasung [29] with the program’s amendment revealing that less than 9000 people in Pasung were being treated [75].

The persistence of Pasung has prompted the Central Government to change the direction of the Free Pasung Program which, at the national level, is now led jointly by the Ministry of Social Welfare and the Ministry of Health. As both Ministries grapple with the enormity of achieving a Pasung-free Indonesia, they continue to set different deadlines. Coordination issues between both ministries revealed a lack of leadership in the government institution on the Pasung issue. According to the Ministry of Social Affairs, Indonesia will be Pasung-free by the end of 2019; however, according to the Ministry of Health this will not be achieved until 2023. Aside from these differing deadlines, the Ministry of Health implements a Free Pasung Program on a more institutional basis to care for persons who have experienced Pasung, while the Ministry of Social Affairs establishes community-based pilot programs with an emphasis on social rehabilitation. The Ministry of Social Affairs is implementing recovery-oriented practices in accordance with their 2013 Social Rehabilitation Program Development Plan, which aims to enable people with mental illnesses who have been incarcerated or experienced homelessness to return to their families as participating and productive citizens while also providing accessible support services for people in their communities [58, 76]. The issue is that the infrastructure to undertake rehabilitation following community treatment is insufficient. In West Java, for example, there is only one such centre, which is 4–5 hours by road away from the West Java Psychiatric Hospital.

Despite this discrepancy in targets, the Ministry of Health has stated that periodic provincial and community reporting on Pasung has begun, with mobile phone and social media technologies being used. At both the national and regional levels, cross-sectoral mental health teams meet on a regular basis to implement the Free Pasung Program. Hundreds of Kader Jiwa (community mental health volunteers) and more than 700 general practitioners and primary health nurses have undergone mental health training and are currently working in the community. Also, 355 general practitioner and nurses from secondary level health care have been trained. There has also been a rise in the number of provinces (10 provinces) which allocated their local fund for mental health and 20 provinces which have Free Pasung Programs at provincial level. Statistical totals derived from national surveys and projections, as well as data monitoring systems, might be inconsistent. Furthermore, because of the inclination for formally freed patients to be returned to Pasung once back in the community, Pasung incidents continue to be documented in Pasung-free areas [59].

A further issue is that, while the overall success in terms of human resource improvement is encouraging, policies and their operationalisation lack family involvement, even though almost every policy made includes the need for community engagement. Article 85 of the Mental Health Act No.18 of 2014 [15], for example, allows communities to participate in mental health treatment by reporting any violence against persons with mental illness or if the person needs assistance. Apart from this commitment, what should a family do if an ill family member exhibits aggressive behaviour and the family’s resources for treatment are limited? The family’s perception of risk and safety from violence is crucial, as earlier research had shown that aggressive behaviour and the family’s incapacity to de-escalate it led to the use of Pasung [10, 77–79]. As a result of caring for the person in Pasung, the family members face stress and felt powerless, believing they have to shoulder the burden alone [55, 80, 81]. Pasung is frequently attributed to the family’s failure to give adequate treatment as a result of the complex policy issues, with the expectation that the family understands how to manage the people with mental illness and avoid Pasung [20, 82]. Furthermore, adequate policy implementation, including consumer and caregiver involvement in ongoing primary care support, has yet to take place, with little or no explanation as to how to operationalize this cooperation in the longer term. In addition to consumer and caregiver involvement, it has been determined that implementation should be aligned with improved coordination among all stakeholders, including health professionals, non-health professionals such as those in social affairs, non-government organisations, and the public at large [62, 83]. Overall, based on policies identified, none of these policies give clear models for the Free Pasung Program teams, and also guidance for safety intervention for the family who is in jeopardy when the person has relapse episodes (see Mental Health Act 1966 [44], Mental Health Act 2014 [15], West Java Regional Legislation 2018 [61]). This policy analysis suggests that Pasung is increasingly being seen as a social problem by the Indonesian government, but that change in practice is slow. There are many complexities involving culture and social context they must be better understood, if policy aiming to address Pasung is to achieve its goals, as concluded by a recent systematic review that sought to understand the use of Pasung [9].

This study has a number of limitations. Results cannot be extrapolated to other parts of Indonesia since West Java is a particularly well-resourced province in an area noted for its progressive mental health policy. Also, we did not include policies at a more provincial level as these are many and varied across the provinces and should be the subject of more focused research. A further limitation relates to the secondary nature of some of our data as we did not expressly ask government officials for further details of existing policies and planned policy reforms. Our conclusions are therefore provisional, and more study is needed to corroborate our findings.

## Conclusion

The effectiveness of polices targeted at releasing mentally ill people from Pasung was assessed in this research. The Mental Health Act of 2014 [15], as well as other health regulations and the overall enhancement of mental health services, have helped to elevate mental health, particularly Pasung, to being a priority on the national health agenda. Unfortunately, more and better policies will not be enough to deliver mental health services to the entire population, especially at the community level where Pasung has tended to persist. Despite implementation efforts such as specific regulations and more operational programs implemented by a few provincial governments in Indonesia, they are not evenly distributed and are more focused on curative and rehabilitative efforts with very minimal focus on prevention. To summarise, Indonesian Pasung health policies and strategies, particularly those in West Java province, lack the clarity and direction needed to implement existing evidence-based treatments ‘in-place’ in the communities where they need to work directly with families to prevent a chronically high burden of Pasung. Failure to obtain continuing treatment and support, in particular, tends to contribute to despair among the actors involved in the Free Pasung Program. This, along with a perception of persons with mental illness as a threat to safety and economic stability for the community, obstructs attempts to eradicate Pasung.

## Electronic supplementary material

Below is the link to the electronic supplementary material.

**Additional file 1:** The Achievements of the Indonesia Free Pasung Program

## Data Availability

All data generated or analysed during this study are included in the published article [and its supplementary files].

## List of Abbreviations

CRPD: Convention on the Rights of People with Disabilities
GSP: Gerakan Stop Pemasungan, The Indonesian Ministry of Social Welfare program to stop Pasung
MDGs: Millennium Development Goals
ODGJ: Indonesian term for people with mental illness
ODMK: Indonesian term for people with mental problems
PIS-PK: Healthy Indonesia Program through Family Approach (Program Indonesia Sehat dengan Pendekatan Keluarga)
SDGs: Sustainable Development Goals
SR: Seclusion and Restraint
WHO: World Health Organization
