# Supplementary material for: Indonesia free from pasung: a policy analysis
Source: Int J Ment Health Syst. 2023 May 3;17:12. doi: 10.1186/s13033-023-00579-6 (PMC10155453; doi:10.1186/s13033-023-00579-6)
Supplement: Supplementary file 1 — Additional file 1: The Achievements of the Indonesia Free Pasung Program [file 13033_2023_579_MOESM1_ESM.docx]

**Supplementary File 1: The Achievements of Indonesia Free Pasung Program**

| **No** | **Year** | **Case Detected** | **Case Intervened (referred or treated at Mental Hospital/psychiatry unit)** |
| --- | --- | --- | --- |
| 1 | 2009 | 213 | 170 |
| 2 | 2010 | 218 | 210 |
| 3 | 2011 | 1258 | 1117 |
| 4 | 2012 | 1582 | 1205 |
| 5 | 2013 | 1783 | 1711 |
| 6 | 2014 | 1637 | 1571 |
| 7 | 2015 | 1999 | 1977 |
|  | Total | 8690 | 7961 |

Source: Directorate of Mental Health Ministry of Health Indonesia based on Health District Report 2015
